# The effect of intranasal oxytocin on social reward processing in humans: a systematic review

**DOI:** 10.3389/fpsyt.2023.1244027

**Published:** 2023-09-14

**Authors:** Jakub Kraus, Eliška Výborová, Giorgia Silani

**Affiliations:** ^1^Department of Clinical and Health Psychology, Faculty of Psychology, University of Vienna, Vienna, Austria; ^2^Department of Psychology, Faculty of Arts, Comenius University in Bratislava, Bratislava, Slovakia; ^3^Department of Psychology, Faculty of Social Studies, Masaryk University, Brno, Czechia; ^4^Faculty of Psychology and Neuroscience, Maastricht University, Maastricht, Netherlands

**Keywords:** oxytocin, social reward, anticipation, consumption, social motivation, wanting, liking, fMRI

## Abstract

Understanding the neurobiology of social reward processing is fundamental, holding promises for reducing maladaptive/dysfunctional social behaviors and boosting the benefits associated with a healthy social life. Current research shows that processing of social (vs. non-social) rewards may be driven by oxytocinergic signaling. However, studies in humans often led to mixed results. This review aimed to systematically summarize available experimental results that assessed the modulation of social reward processing by intranasal oxytocin (IN-OXY) administration in humans. The literature search yielded 385 results, of which 19 studies were included in the qualitative synthesis. The effects of IN-OXY on subjective, behavioral, and (neuro)physiological output variables are discussed in relation to moderating variables—reward phase, reward type, onset and dosage, participants’ sex/gender, and clinical condition. Results indicate that IN-OXY is mostly effective during the consumption (“liking”) of social rewards. These effects are likely exerted by modulating the activity of the prefrontal cortex, insula, precuneus, anterior cingulate cortex, amygdala, and striatum. Finally, we provide suggestions for designing future oxytocin studies.

**Systematic review registration:**

https://www.crd.york.ac.uk/prospero/display_record.php?ID=CRD42021278945, identifier CRD42021278945.

## 1. Introduction

Rewards in general, and social rewards in particular, are salient stimuli, events, objects, or situations that induce approach and consummatory behavior (1). Over the last decades, research in animals has shown that the processing of rewarding stimuli is characterized by (at least) two main components, with partially different neurochemical regulations: (1) “wanting”, i.e., the motivation to mobilize an effort to obtain an *anticipated* reward, subtended by the dopaminergic system (2); and (2) “liking”, i.e., the hedonic response evoked by its *consumption*, subtended, among others, by the opioidergic system (3). While such neurochemical regulation has been extensively documented for primary nonsocial rewards such as food, the neurochemistry of social reward processing is less conclusive, especially in humans. For example, evidence of the involvement of the dopamine and opioids during wanting or liking of social rewards is controversial (4–6), suggesting that different neurochemical systems may play a bigger role in the processing of rewards of social nature, with the most promising candidate being oxytocin (7–10).

Oxytocin is a neuropeptide synthesized in the supraoptic and paraventricular (PVN) nuclei of the hypothalamus and is released both as a hormone to the peripheral system and as a neurotransmitter/neuromodulator into the brain (11). The oxytocin-synthesizing magnocellular neurons project to regions relevant for social behavior and reward processing (12–15), including the amygdala, striatum, ventral pallidum, and the prefrontal cortex (PFC) (16, 17). Oxytocin strongly modulates sociality in rats (9), mice (18), and monkeys (10). In humans, it has been associated with social attachments, trust facilitation, social memory, and fear reduction (8), and the processing of social rewards (19, 20).

It has been suggested that wanting of social rewards is regulated by an interconnected oxytocin-dopamine pathway in the brain (16, 21–23), due to animal evidence of overlapping distribution of oxytocinergic and dopaminergic receptors in both dorsal and ventral striatum, ventral pallidum, and ventral tegmental area (VTA) (24, 25). Furthermore, the oxytocin system seems to interact with the opioid system in the nucleus accumbens (NAcc) and arcuate nucleus to modulate the pleasure derived by its consumption (7, 26–29).

Overall, these findings indicate an important role of oxytocin in the processing of social rewards, presumably by modulating the action of dopamine and opioids. However, findings in humans have been less consistent than in other species, and recent well-powered studies reported no or negative effects of oxytocin administration on various social behaviors (30–32) and, more specifically, on the processing of social rewards (33–37).

Apart from the possibility of oxytocin not being involved in humans to the same extent that in other animals, the inconsistencies in the literature might be due to several reasons:

1. Reward phase: Given that wanting and liking (respectively associated with anticipation and consumption) are neurochemically partially distinct components of reward processing, oxytocin modulation can vary depending on the observed phase.
2. Type of stimuli: The definition of socially rewarding stimuli is very heterogeneous between studies, spanning from real social interactions (38, 39) to pictures of smiling faces (40, 41). Additionally, stimuli are usually not controlled for sex/gender effects, even though it has been repeatedly reported that participants might perceive an opposite-sex/gender stimulus as more rewarding (42).
3. Administration route and dosage: Due to the lower side effects compared to intravenous administration and higher efficiency in elevating cerebrospinal fluid (CSF) concentrations (43), oxytocin is mainly administered intranasally (15, 44). The commonly administered dose is 24 international units (IU), but there is little evidence of such dose being the most effective (45). For example, a study by Cardoso et al. used 24 and 48 IU in healthy individuals and found stress-attenuating effects only in the group with 24 IU, suggesting that a higher dose might overstimulate the oxytocin system resulting in no effects (46). Other authors argue for the greatest efficacy of even lower dosages of 8 IU of oxytocin to promote social functioning (45, 47, 48). These findings are broadly consistent with the hypothesized inverted U-shaped dose–response of oxytocin (37, 49). Here, lower levels of administered oxytocin (e.g., 8 or 24 IU) should theoretically move the concentrations of oxytocin in the system closer to the peak, whereas higher doses (e.g., 40 IU) can overstimulate the oxytocin system and result in no effect (50).
4. Participants’ sex/gender: lower baseline levels of endogenous oxytocin in men compared to women have been previously documented (51). Recent findings also suggest sex-dependent regulation of social rewards by oxytocin (52), with some studies observing opposite effects on brain activity during consumption of social rewards (37, 53). The oxytocin effects on women might further be biased by the usage of oral contraceptives (54). Notably, in spite of its relevance, sex/gender effects on the neurochemical regulation of social reward processing in humans, are largely unknown.
5. Onset time: Following single intranasal oxytocin administration, the time the participant has to wait, can also affect oxytocin concentrations in the system (55).
6. Clinical condition: Administration of oxytocin may have different effects on social reward processing in clinical populations and healthy controls. Research for example shows that traumatic experiences [e.g., in post-traumatic stress disorder (PTSD) or depressive states] may affect hormonal systems resulting in reduced production and release of oxytocin (56). In such cases, oxytocin administration could result in more profound effects as compared to participants with normal endogenous levels of oxytocin.

Given the inconsistencies in the effects of oxytocin in modulating social reward processing in humans (31), it is therefore crucial to consider methodological differences. To this aim, we reviewed human empirical studies that utilized intranasal oxytocin to assess social reward processing and systematically summarized their results in relation to the aforementioned points. We also assessed the quality of each individual study, including potential problems to detect small effects due to insufficient sample size. The findings will ultimately help to evaluate the role and the effectiveness of intranasal oxytocin in regulating social reward processes and provide further suggestions for designing oxytocin studies in the field of social reward.

## 2. Methods

This review is based on the PRISMA guidelines (Preferred Reporting Items for Systematic Reviews and Meta-Analyses) (57) and is publicly pre-registered in PROSPERO (International prospective register of systematic reviews). Pre-registration information is available on the following link.^1^ The quality of the studies, including reporting, external/internal validity, and power, was assessed with the widely accepted tool for quality assessment developed by Downs and Black (58). The quality assessment was performed by one reviewer, and the decision was checked by a second reviewer. Details on the assessment are provided in the Supplementary material.

### 2.1. Search strategy and exclusion criteria

Three major databases—Web of Science, Scopus, and Pub-Med, and three preprint servers—BioRxiv, MedRxiv, and PsyArXiv, were searched from September 2021 until May 2022 (see Supplementary material for the full search strategy including all the databases and preprint servers utilized). The keywords were combined into command lines for individual databases and ordered to appear in the title or abstract of the empirical papers. The same following search terms were used for all databases combined with Boolean operators AND and OR: oxytocin*, intranasal*, *social*, affilia*, reward*, wanting, incentive*, goal* pursuit, motivat* salien*, desir*, liking, hedonic impact, hedonic value, hedonic react*, pleasur*, and approach* motivat*.

**Figure 1:**
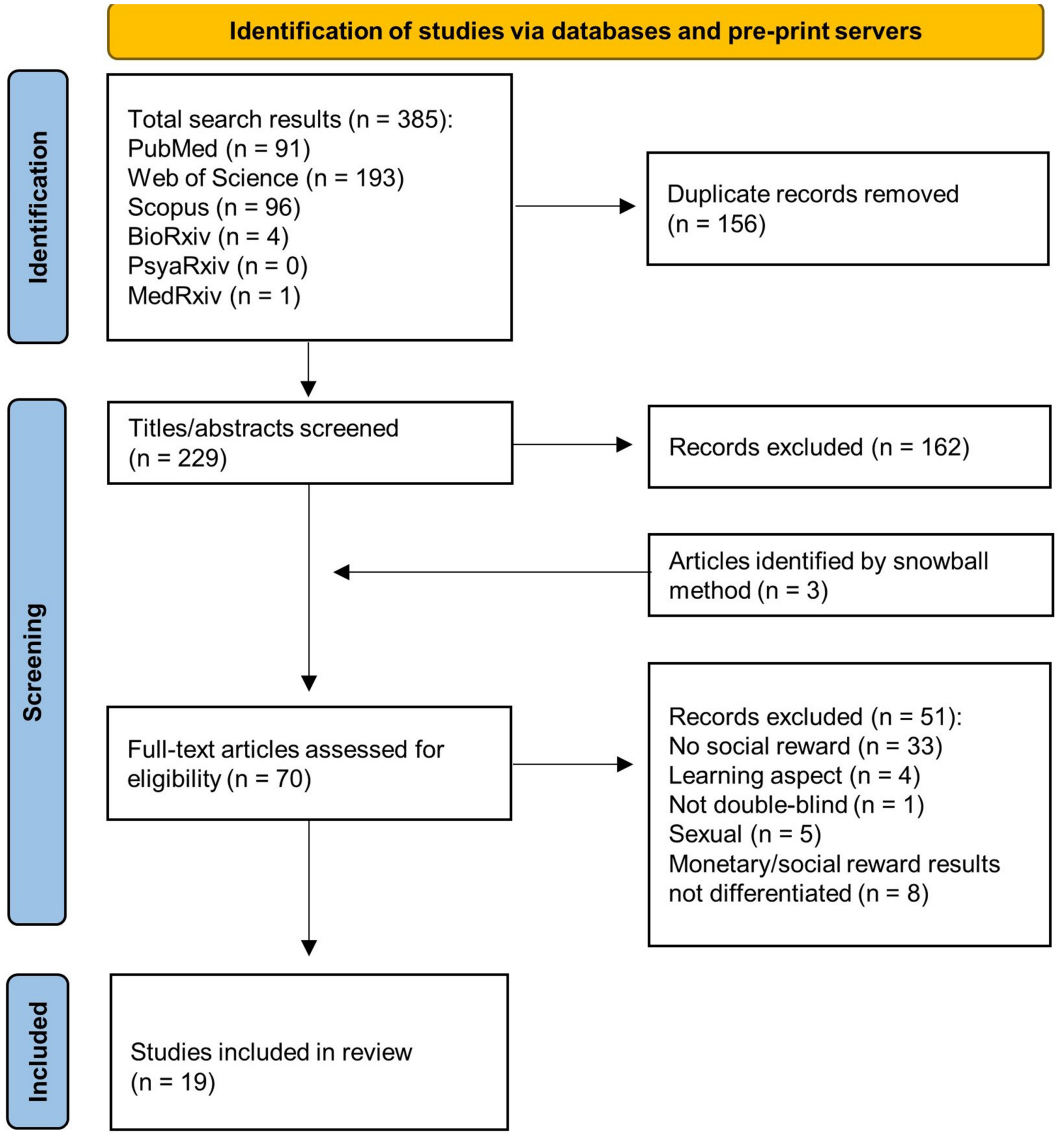
PRISMA flow diagram illustrating the search process.

The selection process of the relevant studies is depicted in the PRISMA flow diagram (Figure 1). Only randomized, double-blind, placebo-controlled human studies, utilizing intranasal oxytocin and social reward tasks were included in the review. For wanting, the measurement had to occur during the anticipation phase; any behavioral or subjective measures had to reflect motivation/desire to get the reward. For liking, the measurement had to occur during the consumption phase; any behavioral or subjective measures had to reflect positive feelings derived from the reward receipt.

Exclusion criteria were: (a) social reward stimuli are not distinguishable from non-social rewards; (b) wanting and/or liking components are not assessed [instead, e.g., main focus on learning, i.e., predictive associations and cognitions (59)]; (c) studies including no behavioral/fMRI measures; (d) social rewards are represented by sexual stimuli, as affiliative and sexual reward may be subtended by different neurochemical systems (60, 61). Opposite gender stimuli were not generally considered as sexual rewards unless they were of sexual character or were used to measure some form of sexual arousal.

### 2.2. Synthesis of outputs and results

The synthesis of results follows a narrative approach. To address the main aim of the review, the relevant outputs of all included studies were first summarized into the following sections (see Table 1): (1) general information (Clinical condition, Sex/Gender, Oral contraceptives, Sample size, and Age), (2) the paradigm and the task used (Task, Task type, Social reward, and Experimental paradigm), and (3) the type of measures and outcome variables (Reward Component, Measures, Analysis, and Results). A more detailed description of each task is reported in Table 2 and brain regions of interest (ROIs) modulated by oxytocin administration in Table 3. Results are discussed based on the following classification criteria: (a) Reward phase (anticipation, consumption), (b) Type of stimuli (simple, i.e., non-interactive/unimodal, interactive), (c) Dosage, (d) Participants sex/gender (female, male); and (e) Clinical condition (clinical, healthy population).

**Table 1:** Result of studies included in the systematic review.

Study	CC	Sex/Gender	OC	Sample size			Age (M ± SD)	Task	Task type	Social reward	Experimental paradigm			Reward phase	Measures	Analysis	Results
				*N*	OXY	PLC					Dose (IU)	Onset (mins)	Design				
Alvares et al. (62)	HC	M,F	Controlled, no change	36(?M)	18(?M)	18(?M)	21.91 ± 5.93	Cyberball task	Interactive	Social inclusion	24	45	BS	Anticipation	Self-reported	Desire to play again with the includers—two visual analog scales	Greater desire to play the game again after OXY vs. PLC. No significant differences when female participants excluded
															Behavioral	Number of button presses	No drug effect
Groppe et al. (63)	HC	F	0/28	28	14	14	26.64 ± 5.55	Social incentive delay task	Simple stimulus	Picture of a happy face	approx. 26	30	BS	Anticipation	Behavioral	Reaction times	No main effect or interaction with treatment group was observed
															fMRI	ROI: VTA	↑ VTA
Xu et al. (64)	HC	M,F	0/37	77(40 M)	39(20 M)	38(20 M)	22.5 ± 0.4 (PLC), 22.1 ± 0.3 (OXY)	Cyberball	Interactive	Social inclusion	40	45	BS	Anticipation	Self-reported	Desire to play again with the includers	OXY increased the desire to play again with the excluders and decreased the preference for includes. OXY effect was present only for males
Fulford et al. (65)	HC, SCZ	M,F	?	85(60 M): 43HC(29 M), 42SCZ(31 M)	85(60 M)		41.3(HC), 42.74(SCZ)	Social vigor task	Interactive	Encouraging + positive evaluations	40	30	WS	Anticipation	Behavioral	Average key presses per second	In the social reward condition, the effect of OXY did not survive *post hoc* pairwise comparisons, nor it did interact with sex or MHS
Bradley et al. (38)	HC, SCZ	M	–	88(51HC)	48(26HC)	40 (25HC)	46.23 ± 13.6 (PLC); 40.47 ± 12.23(OXY)	Auction game	Interactive	Social status of a winner	40	30	BS	Anticipation	Behavioral	Overall decrease in bidding throughout the game (drug*trial)	Sign. effect of OXY: Participants on PLC decreased their bids over time, whereas participants on OXY did not
Wang & Ma (66)	HC	M	–	56	56		21.21 ± 2.76	Pay-to-know choice task	Interactive	Positive social evaluation	24	35	WS	Anticipation	Behavioral	The amount of money participants were willing to forgo	The subjective value of positive social evaluation was not generally affected by oxytocin
Nawijn et al. (67)	HC, PTSD	M,F	8/18 HC, 7/14 PTSD	72(41 M), 37 HC trauma-exposed (19 M), and 35 PTSD (21 M)	72(41 M), 37 HC trauma-exposed (19 M), and 35 PTSD (21 M)		M: HC: 41.11 ± 10.86, PTSD: 42.29 ± 9.83, F: HC: 38.06 ± 9.08, and PTSD: 38.21 ± 9.85	Social incentive delay task	Simple stimulus	Happy face image	40	50	WS	Anticipation	fMRI	WB ROI: putamen, caudate, AMY, and AI	No drug effect, main or interaction with PTSD status or sex
															Behavioral	Reaction times (RTs), relative reaction times (RRTs), i.e., RTs during reward relative to neutral trials	Oxytocin did not significantly affect RT or RRT
														Consumption	fMRI	WB ROI: striatum (putamen + caudate), AMY, anterior insula	WB: No main effects of drug or interaction with sex. ROI: PTSD: ↑ R putamen, L anterior insula I, HC: ↓ R putamen, L anterior insula
												2 h			Self-reported	Perceived rewardingness of the happy faces on a five-point Likert scale (0: Not at all—5: Very much)	Oxytocin did not significantly affect subjective ratings
Greene et al. (35)	ASD	M,F	?	28 (26 M)	28 (26 M)		13.43 ± 2.36	Social incentive delay task	Simple stimulus	Pictures of smiling faces	24	?	WS	Anticipation	Behavioral	Reaction times	No drug effect
															fMRI	WB ROI: SFG., MFG, OFC., paracingulate gyrus, AMY, NAcc, insula, thalamus, caudate, ACC, putamen PPI: R NAcc, and L ACC	No drug effect
														Consumption	fMRI	WB ROI: SFG., MFG, OFC., paracingulate gyrus, AMY, NAcc, insula, thalamus, caudate, ACC, putamen PPI: R NAcc, and L ACC	WB: ↓ R frontal pole
Ma et al. (39)	HC	M,F	0/51	104(53 M)	49(25M)	55(28 M)	21 ± 0.22	Social sharing task	Interactive	Social sharing	40	45	BS	Anticipation	Self-reported	Three-item subscale of a social sharing questionnaire measuring the intentions to communicate feelings with the other person	No sign. Effect of OXY on the desire to communicate feelings with neither stranger nor friend
														Consumption	fMRI	WB ROI: AMY PPI: insula	WB: ↑M, ↓F: R insula, R PCG (in the friend condition). ROI:↓F, (trend ↑M): AMY (in the friend condition). PPI: ↑M, ↓F: R insula—L AMY (in the friend condition)
Chen et al. (73)	HC	M	–	46	46		21.22 ± 2.77	Touch in the form of a massage	Simple stimulus	Touch in the form of a massage	24	45	WS	Anticipation	Self-reported	Nine-point Likert scale of willingness to pay for the massage	No sign. Effect of OXY on ratings of how much subjects were willing to pay for the massage
														Consumption	Self-reported	Nine-point Likert scale of touch pleasantness	OXY sign. Increased pleasantness ratings
															fMRI	WB	↑OFC, DS, VTA, dACC, insula, mPFC, PCC, precuneus, PHG, AMY, hippocampus, superior and medial temporal regions and IPL, cuneus, fusiform gyrus and occipital gyrus, PCG, cerebellum
Mayer et al. (36)	ASD, HC	M	–	71(35ASD)	71(35ASD)		26.2 ± 4.7(ASD), 27.1 ± 4.4(HC)	Incentive delay task	Simple stimulus	Pictures of smiling faces	24	40	WS	Anticipation	Behavioral	Hit rates (response times)	No drug effect, main or interaction
															fMRI	ROI: ventral striatum WB	No significant effects of OXY or differences between patients and controls
														Consumption	fMRI	WB ROI: AMY	WB: No significant OXY effects, group or group × drug interactions. ROI: ↑single voxel within the L AMY
Ellingsen et al. (68)	HC	M,F	14/20, no sign. Differences	39(19 M)	39(19 M)		26 (range 20–39)	Human gentle touch	Simple stimulus	Human gentle touch	40	40	WS	Consumption	Self-reported	Visual analog rating scale of touch pleasantness	No drug effect.
Scheele et al. (42)	HC	M	–	40	40		25.75 ± 3.82	Human gentle touch	Simple stimulus	Human gentle touch	24	30	WS	Consumption	fMRI	WB ROI: OFC, pACC	WB: ↑ mid and anterior insula ranging to the OFC, PCu, Cu ROI: ↑ bilateral OFC, pACC
															Self-reported	Visual analog scale ranging from 1 (very unpleasant; a sad smiley) to 20 (very pleasant, a happy smiley)	OXY increased the pleasantness of female touch, No sign. Effect of OXY on pleasantness ratings during the male touch condition
Hu et al. (69)	HC	M	–	54	?	?	19.86 ± 1.49	RALT task -feedback phase	Simple stimulus	Neutral changed to a smiling face	24	45	BS	Consumption	fMRI	WB ROI: bilateral hippocampus, AMY, and PHG	WB: ↑ R medial PFG, PCu
Gregory et al. (70)	HC	F	?	59	?	?	Nulliparous: 23.8 ± 3 post-partum: 30.21 ± 4.4	One-back matching task	Simple stimulus	Pictures of infants	24	30	BS	Consumption	fMRI	ROI: VTA, NAcc	↑VTA
Andari et al. (40)	ASD	M,F	?	20 (19 M)	8	12	26.37 ± 8.45	Cyberball task	Simple stimulus	Social inclusion	24	40–45	BS	Consumption	fMRI	WB ROI: OFC, caudate	WB: ↑ R mid orbital gyrus, ACC, ROI:↑ R OFC
Gordon et al. (71)	ASD	M,F	?	16 (13 M)	16 (13 M)		13.67 ± 2.76	Affective Voices task	Simple stimulus	Listening to a happy voice	16–19 years: 24; 12–15 years: 18; 7–11 years: 12	?	WS	Consumption	fMRI	WB PPI: NAcc, AMY	L NAcc—PCu, Cu, L aSMG/HG, R temporo-occipital FFG R NAcc—PCu, R MFG, L planum temporale/aSMG, intra-calcarine cortex, R angular gyrus. AMY—posterior occipital regions. L AMY—occipital pole, R AMY—PCu
Bos et al. (33)	HC	F	23/23	23		23(F)	20.2 ± 1.4	Infant face task	Simple stimulus	Picture of a cute infant face	24	49	WS	Consumption	Self-reported	Self-report of infant face cuteness	No drug effect.
															fMRI	WB ROI: AMY, putamen, caudate, insula, ACC, VTA, and NAcc	ROI: ↓R AMY, ↓R putamen, ↓VTA
Riem et al. (72)	HC	F	30/42, included as a confound regressor	42	22, 20		28.71 ± 6.93	Infant auditory stimuli	Simple stimulus	Infant laughter	24	40	–	Consumption	fMRI	WB, ROI:AMY, NAcc, IFG, insula, PPI: AMY	ROI: ↓AMY PPI: R AMY – left OFC, hippocampus, left Pcu, R angular gyrus, MTG, L ACC

CC, Clinical condition; HC, Healthy control; ASD, Autism spectrum disorder; SCZ, Schizophrenia; PTSD, Post-traumatic stress disorder; M, Male, F, Female; ↑, Increase, ↓, Decrease; —, Functional connectivity; R, Right; L, Left; OC, Oral contraceptives; OXY, Oxytocin; PLC, Placebo; IU, International unit; WS, Within-subject; BS, Between-subject; WB, Whole-brain; ROI, Region-of-interest; PPI, Psychophysiological interactions (functional connectivity); ACC, Anterior cingulate cortex; pACC, Posterior anterior cingulate cortex; PCC, Posterior cingulate cortex; OFC, Orbitofrontal cortex; DS, Dorsal striatum; mPFC, Medial prefrontal cortex; vlPFC, Ventral lateral prefrontal cortex; STS, Superior temporal sulcus; MFG, Middle frontal gyrus; SFG, Superior frontal gyrus; MTG, Middle temporal gyrus; PHG, Parahippocampal gyrus; IPL, Inferior parietal lobule; PCG, Post-central gyrus; PCu, Precuneus; Cu, Cuneus; FFG, Fusiform gyrus, aSMG, Anterior supramarginal gyrus; SMA, Supplementary motor area; NAcc, Nucleus accumbens; and VTA, Ventral tegmental area.

**Table 2:** Description of tasks used in the included studies.

Task	Description	Study
Cyberball task	Ball tossing game with virtual players (thought real), the participants experienced either social reciprocity (sending the ball equally to all) or exclusion (sending the ball only to the other players and not the participant). Social reward was measured during social reciprocity phase.	(40, 62, 64)
Auction game	35-trial Auction Game that quantifies preferences for monetary vs. social reward—bidding against five other virtual players (thought real) to win virtual items each with a true common value. Participants are provided with the best strategy to maximize their profit: Risk Neutral Nash Equilibrium ($25 if the range is $25–35), there is no other strategy that would yield a higher payoff. Social reward is the status of a winner (highest bidder).	(38)
Social vigor task	The task measures effort exertion for reward in the context of social encouragement. Points accumulated on the screen as participants rapidly pressed the key. In half the trials, trained research assistants (RAs) delivered a standard set of positive statements (e.g., “Good,” “Keep going!” “Awesome. You’re doing great!”). Social reward derived from the positive statements during social encouragement.	(65)
Social sharing task	Social sharing: viewing a picture together with a friend/stranger sitting in another room. To provide a context for the “stranger” condition, subjects were instructed that another pair of friends (strangers) underwent the experiment simultaneously in a different room. Social reward derived from the social sharing situation.	(39)
Pay-to-know choice task	Participants provided their own photo and a self-introduction essay including their name, age, personality traits, likes/dislikes, hobbies, and interests. Then they were told that other people on the computer program would make evaluations. Participants were presented with a trait word and the evaluation source (the face of a stranger), and then asked to choose between a “to-know” option and a “not-to-know” option associated with different amounts of tokens.	(66)
Human gentle touch	Human gentle touch: 3 s duration soft strokes with a velocity of approximately 5 cm/s., administered between two parallel areas (each about 15 cm long) of the left forearm by a research assistant wearing a silk glove. Social reward derived from the sensory stimulus pleasantness.	(68)
Human gentle touch	Touch was administered to the shin and calf of both legs, moving from the knees toward the ankles. A 20-cm zone of the shin was touched during 4 s, with touch velocity of 5 cm/s. A photo of the experimenter was presented during the task. Social reward derived from the sensory stimulus pleasantness.	(42)
Infant face task	The stimuli consisted of nine different pictures of an infant faces (which comprised the normal condition), which were manipulated to create additional low-cuteness and high-cuteness condition of the same face. Participants were instructed to carefully look at the stimuli and, after the offset of the face, use the button box in their right hand to rate whether the presented face was “not very cute,” “cute,” or “very cute.”	(33)
One-back matching task (pictures)	This task was chosen to ensure attention during the presentation of the relevant pictures. Pictures included sexually explicit, crying infant, smiling infant, and neutral photos. Neutral images were taken from the IAPS and sexual and infant images were taken from publicly available websites.	(70)
Infant auditory stimuli	Participants listened to intensity-matched infant crying and infant laughter sounds of the International Affective Digitized Sounds system. Neutral auditory control stimuli were created identical to the original auditory stimuli.	(72)
Touch in the form of massage	Social touch in the form of massage: four massage conditions: social (manual), imagined social, machine massage, and imagined machine massage. Manual foot massage was administered by a professional masseur; the machine massage was applied by a commercial foot massage machine which involved them wearing boots on each foot. Social reward measured as the response to real or imagined manual massage.	(73)
Affective voices task	During Affective Voices, participants listened, eyes shut, to alternating blocks of angry (ANG), and happy (HAP) non-word vocalizations (3 per condition, 15 s each), with listening blocks separated by 15 s silent periods, during which eyes remained shut. Stimuli were taken from the Montreal Affective Voices dataset. Each block contained five utterances (527–1,742 ms) separated by brief silences (1,258–2,473 ms)	(71)
RALT task	This task required subjects to judge the category membership “A” or “B” of three-digit numerical items repeatedly presented on a computer screen, with visual feedback immediately following each item-category judgment. The letters “A” and “B” flanked either a female or a male face, which changed from neutral to happy for correct responses or from neutral to angry for incorrect responses.	(69)
Social incentive delay task	Graphical cues provided information on the type of outcome to be expected after successful (=hit) or unsuccessful (=miss) performance (i.e., hitting a button within a certain time window). In the social reward condition, happy faces were shown if the reaction was performed in time (i.e., hit).	(35, 36, 63, 67)

The tasks described in the upper part of the table represent the interactive tasks in which participants believe to interact with real people. The lower part of the table comprises task using simple social stimuli to generate social reward.

**Table 3:** Region by region significant activations under OXY.

Region	Study	Significance threshold	Site	Peak MNI			*Z*/*t*	Outcome	Voxels
				*x*	*y*	*z*			
PFC	(72)	Cluster-corrected at *p* < 0.05 and *Z* = 2.3	R AMY—L OFC	−44	18	−12	*Z* = 3.32	↑F	185
	(42)	*p* < 0.05 (FWE corrected)	R	38	28	−2	*t* = 4.53	↑M	53
			L	−32	28	0	*t* = 3.03	↑M	31
	(69)	*p* < = 0.0001, uncorrected, cluster size > *n* = 10	R	3	60	3	*t* = 3.81	↑M	16
	(40)	*p* < 0.05 (FWE corrected, voxel-wise *p* < 0.001)	R	6	51	−15	*t* = 8.46	↑M	?
	(71)	Cluster-corrected at *p* < 0.05 and *Z* = 2.3	R NAcc—MFG	30	6	62	*Z* = 3.24	↑M(3F)	432
	(73)	*p* < 0.025 (FDR corrected, cluster > 10 voxels)	R inferior OFC	57	21	−6	*t* = 3.90	↑M	?
			R superior OFC	21	60	6	*t* = 3.76	↑M	?
			L IFG	−54	36	3	*t* = 4.43	↑M	?
			L MFG	−27	−6	51	*t* = 3.51	↑M	?
			R MFG.	39	39	9	*t* = 3.76	↑M	?
			R MFG	33	−6	48	*t* = 3.63	↑M	?
			R dorsal medial PFC	12	18	48	*t* = 3.61	↑M	?
			R medial PFC	9	51	15	*t* = 3.80	↑M	?
Insula	(42)	*p* < 0.05 (FWE corrected)	R	40	4	14	*t* = 4.61	↑M	394
			R anterior	38	24	10	*t* = 4.47	↑M	
	(67)	*p* < 0.05 (FWE corrected)	L anterior	−38	10	14	*Z* = 3.53	↑PTSD, ↓HC	9
	(39)	*p* < 0.05 (FWE corrected)	R	39	−18	12	*t* = 4.32	↑M, ↓F	351
			R insula—L AMY					↑M, ↓F	
	(73)	*p* < 0.025 (FDR corrected, cluster > 10 voxels)	L anterior	−36	24	6	*t* = 3.66	↑M	?
			L posterior	−36	−18	−3	*t* = 3.48	↑M	?
			L middle	−36	3	−3	*t* = 3.39	↑M	?
PCu	(42)	*p* < 0.05 (FWE corrected)	R	20	−82	34	*t* = 4.74	↑M	547
			R	28	−78	28	*t* = 4.16	↑M	
	(69)	*p* < = 0.0001, uncorrected, cluster size > *n* = 10	L	−6	−45	30	*t* = 4.88	↑M	87
			R	3	−33	45	*t* = 4.79	↑M	104
	(71)	Cluster-corrected at *p* < 0.05 and *Z* = 2.3	R AMY—PCu	6	−60	12	*Z* = 3.33	↑M(3F)	1,015
			L NAcc—PCu	6	−52	62	*Z* = 3.52	↑M(3F)	2,825
			R NAcc—PCu	4	−70	50	*Z* = 3.10	↑M(3F)	-
	(73)	*p* < 0.025 (FDR corrected, cluster > 10 voxels)	L	−21	−69	39	*t* = 4.17	↑M	?
			R	27	−63	33	*t* = 4.01	↑M	?
ACC	(72)	Cluster-corrected at *p* < 0.05 and *Z* = 2.3	L AMY—L ACC	−8	10	38	*Z* = 3.70	↑F	172
			R AMY—L ACC	−6	10	38	*Z* = 3.33	↑F	116
	(42)	*p* < 0.05 (FWE corrected)	R	2	38	8	*t* = 3.05	↑M	105
	(40)	*p* < 0.05 (FWE corrected, voxel-wise *p* < 0.001)	?	?	?	?	*t* = 6.35	↑M	?
	(73)	*p* < 0.025 (FDR corrected, cluster >10 voxels)	R	3	33	24	*t* = 5.09	↑M	13,644
AMY	(72)	Cluster-corrected at *p* < 0.05 and *Z* = 2.3	R AMY—left OFC, hippocampus, left PCu, R angular gyrus, MTG, ACC; L AMY—L ACC						
	(71)	Cluster-corrected at *p* < 0.05 and *Z* = 2.3	AMY—posterior occipital regions; L AMY—occipital pole; R AMY—PCu					↑M(3F)	
	(39)	*p* < 0.05 (FWE corrected)	L	−21	−9	−12	*t* = 3.61	↓F, (↑M)	2
			R	21	−9	−15	*t* = 3.39	↓F, (↑M)	2
			R insula—L AMY	−24	−9	−15		↑M, ↓F	
	(73)	*p* < 0.025 (FDR corrected, cluster > 10 voxels)	R	24	−3	−15	*t* = 3.07	↑M	?
Striatum	(70)	*p* < 0.05 (FDR corrected)	VTA	?	?	?	?	↑F	?
	(71)	Cluster-corrected at *p* < 0.05 and *Z* = 2.3	L NAcc—PCu, Cu, L aSMG, R FFG; R NAcc—PCu, R MFG., L planum temporale, intra-calcarine cortex, and R angular gyrus						
	(67)	*p* < 0.05 (FWE corrected)	R putamen	26	10	8	*Z* = 3.93	↑PTSD, ↓HC	29
	(73)	*p* < 0.025 (FDR corrected, cluster > 10 voxels)	R caudate	21	15	18	*t* = 4.31	↑M	?
			R putamen	15	3	9	*t* = 4.10	↑M	?
			R caudate	15	18	6	*t* = 3.68	↑M	?
			L putamen	−21	−9	6	*t* = 4.26	↑M	?

PTSD, Post-traumatic stress disorder; M, Male; F, Female; ↑, Increase; ↓, Decrease; —, Functional connectivity; R, Right; L, Left; OXY, Oxytocin; ACC, Anterior cingulate cortex; PFC, Prefrontal cortex; OFC, Orbitofrontal cortex; NAcc, Nucleus accumbens; AMY, Amygdala; NAcc, Nucleus accumbens; MFG, Middle frontal gyrus; IFG, Inferior frontal gyrus; FFG, Fusiform gyrus; aSMG, Anterior supramarginal gyrus; FWE, Family-wise error; FDR, False discovery rate.

## 3. Results

Nineteen studies were identified, based on the search strategy and exclusion criteria (see Figure 1). The identified studies encompassed together 984 participants of age ranging from 13 to 43. The sample size varied from 16 to 104. Five of the studies were conducted in China (39, 64, 66, 69, 73), and in the United States (35, 38, 65, 70, 71), three in Germany (36, 42, 63) and in Netherlands (33, 67, 72), one in Norway (68), Australia (62), and France (40). All of the studies have been published within the last decade. The overall risk of bias across all studies was assessed as mainly low (see details on the Quality assessment in the Supplementary material).

### 3.1. Reward anticipation

Eleven studies measured social reward anticipation (35, 36, 38, 39, 62–67, 73), out of which eight used behavioral measures (38, 65), four studies used fMRI measurement (35, 36, 63, 67), and four used self-reported measures (39, 62, 64, 73).

Only one behavioral study (38), and two studies using self-reported measures (62, 64), found significant effect of oxytocin.

On the fMRI level, four studies measured anticipatory social reward, all of which used ROI analysis (35, 36, 63, 67), three used whole-brain analysis (35, 36, 67), and one measured functional connectivity (35). Only one study found a significant effect of oxytocin treatment (63), which was conducted on female participants and precisely, increased VTA activity during social reward anticipation after the treatment.

### 3.2. Reward consumption

Thirteen studies measured social reward consumption (33, 35, 36, 39, 40, 42, 67–73), out of which only one did not use fMRI measurement (68), but used self-reported measures; four studies used both these measurement types (33, 42, 67, 73). Overall, nine out of 13 studies found significant effects of IN-OXY on social reward consumption on at least one level of measurement, and specifically an enhancement of consummatory social reward processing after IN-OXY. Three studies found also decreases in neural processing in female participants (33, 39, 72). In the following paragraphs, results will be summarized according to individual levels of measurement.

For self-reported measurement, three studies used social touch stimulus as a form of social reward, one used smiling faces. Of these, two found significant effects of oxytocin on social reward consumption, which was measured as the level of the pleasantness of a massage (73) or gentle human touch (42) (see Table 1). One study measured self-reported cuteness of the shown infant pictures with null results (33).

Eleven out of 12 fMRI studies found significant effects of IN-OXY on social reward processing, only one reported no oxytocin effects (36). Of the 12 fMRI studies, five studies found effects on the whole-brain level, three of them report effects in PFC (40, 69, 73), two report effects in the insula (39, 73), precuneus (69, 73), and ACC (40, 73). Ten studies used region-of-interest (ROI) analysis with predefined target regions in the brain. 3/4 studies found significant effects in the PFC (40, 54, 72), 1/2 in the insula (67), 2/3 in the ACC (42, 72), 2/4 in the striatum (67, 70), and 2/6 in the amygdala (39, 72). Of the four fMRI studies measuring effective or functional connectivity (35, 39, 71, 72), three found significant effects of IN-OXY (39, 71, 72). Ma et al. (39) between the right insula and left amygdala, Gordon et al. (71) found complex changes in connectivity between the subcortical sites of NAcc and amygdala, and the prefrontal cortices. Finally, Riem et al. (72) found connectivity between the amygdala and vast network of regions including left OFC, hippocampus, precuneus, angular gyrus, MTG, and ACC.

Overall, of the 12 studies measuring the effects of IN-OXY on the neural processing of social reward consumption, changes in the activity or functional connectivity were reported six times in the PFC (40, 42, 69, 71–73), four times in the insula (39, 42, 67, 73), precuneus (42, 69, 71, 73), four times in the amygdala (39, 71–73), striatum (67, 70, 71, 73), and ACC (40, 42, 72, 73). Specifics of these results are detailed in Table 3.

### 3.3. Type of stimuli

Seven studies used an interactive task to generate social reward (38–40, 62, 64–66), and 12 used a positive social stimulus without a preceding social interaction (see Table 1). Of these, three studies used a touch stimulus (42, 68, 73), of which one paired it with a face stimulus (68), and one administered touch in the form of a massage (73). Five studies used pictures of smiling faces (35, 36, 63, 67, 69), of which one used a video of neutral face changing into a smiling face (69). Two studies used pictures of infant faces (33, 70). One study used a stimulus of a happy voice (71) and one study used infant laughter (72).

A significant effect of oxytocin was found in eight out of 12 studies using simple social stimuli (54, 63, 67, 69, 71, 73), where only once the effect was related to reward anticipation (63). A significant effect of oxytocin treatment was further shown in total in five out of seven studies using the interactive task (38–40, 62, 64) of which the effect was found in both studies that measured reward consumption (39, 40).

### 3.4. Dosage

Six studies administered 40 IU IN-OXY (38, 39, 64, 65, 67, 68), of which four found significant effect of OXY (38, 39, 64, 67). Thirteen out of 19 studies used a dose of approximately 24 IU IN-OXY. Specifically one study used approximately 26 IU (63), one study used age-dependent dosing ranging from 12 to 24 IU (71), and 11 studies used exactly 24 IU (33, 35, 36, 40, 54, 62, 66, 69, 70, 72, 73). Only one of these studies did not find a significant effect of treatment of any kind (36).

### 3.5. Participants’ sex/gender

Six studies included only men in their sample (36, 38, 42, 66, 69, 73), four studies measured only women (33, 63, 70, 72).^2^ The other 10 studies included both men and women; but one of these included only one female participant out of 20 (40), another study included only two women out of 28 (35), and another only three women out of 13 (71); therefore, we will further refer to these studies as if they were conducted solely on men. Finally, one study did not report the exact number of women in the sample for social reward measurement (62). Thus, in total, seven studies included a significant portion of both male and female participants. The study by Ma et al. (39) reported a significant difference between men and women where social reward processing was downregulated by oxytocin in women, while upregulated in men. Another study reported a significant oxytocin effect for men while no effect for women (64). Two studies reported significant effects of oxytocin regardless of gender (62, 67), and two studies reported no effects of treatment regardless of gender (65, 68). Finally four studies used a solely female sample and found significant oxytocin effects (33, 63, 70, 72), however two of these studies found also an effect in opposite direction (decrease in activity), and this was mainly in the amygdala (33, 72).

Eight studies reported information about the usage of oral contraceptives (33, 39, 62–64, 67, 68, 72), three of these reported that no women in their sample used oral contraceptives (39, 63, 64), three studies controlled for the effect (62, 68, 72), one study enrolled only participants using oral contraceptives (33), and one other study reported that the number of women using and not using oral contraceptives was balanced between the patient groups (67).

### 3.6. Onset time

Five studies set the onset of the experiment 45 min after the IN-OXY administration (39, 62, 64, 69, 73), all reporting significant effects of OXY against placebo at least on one level of analysis (subjective, behavioral, and fMRI). Three studies used shorter onset time of 40 min (36, 68, 72), one of which showed no significant results (36). Another study used an unspecified onset within 40–45 min after administration, with significant findings on brain activity (40). Six studies had onsets around 30 min (38, 54, 63, 65, 66, 70), of which one was of 35 min (66). Four of these studies reported significant effect of IN-OXY manipulation (38, 54, 63, 70). Two studies utilized an onset of around 50 min, both reporting significant findings but only on brain activity (33, 67). Lastly, one study did not report the onset time at all (71).

### 3.7. Clinical condition

Twelve studies used only healthy participants (see Table 1), four studies used patients with autism (35, 36, 39, 71), of which one compared them with healthy controls (36), two studies compared schizophrenia patients with healthy controls (38, 65), and one study compared PTSD patients with healthy trauma-exposed participants (67). Overall, 12 out of 16 studies using healthy participants found an effect of oxytocin treatment (33, 35, 39, 42, 67–73), three out of four studies using autistic patients found a significant effect (35, 40, 71), and the only study using PTSD patients found a significant effect as well (67).

## 4. Discussion

The current review employed a systematic narrative approach to identify the effects of intranasal oxytocin on social reward processing. Importantly, this review differentiated between the anticipatory and consummatory components of social reward and considered different aspects that may affect the action of OXY on social reward processing. The results provide evidence that intranasal oxytocin can effectively modulate social reward processing, even though its effects are mainly detectable on the neural level. A consistent pattern was found for the effects of oxytocin during the consumption phase; however, the effects during anticipation were less prominent.

### 4.1. Social reward anticipation

For the anticipatory phase, half of the studies using *self-reported measurement* found a significant IN-OXY effect (62, 64). However, in the study by Xu et al. oxytocin significantly increased the desire (i.e., wanting social reward) to play the game again with the excluders and not the includes, which was considered to generate social reward (64). The plausible explanation is that this study was conducted in a collectivistic culture (i.e., community prioritizing the group over the individual), where restoring relationships with the community might be more relevant than individual pleasure. It is also of interest that both of these studies utilized the same type of paradigm (cyberball task) as compared to the studies using social touch and social sharing paradigms (39, 73) that found no such effects on self-reports. Nearly none of the *behavioral studies* found an effect of oxytocin. Apart from oxytocin not being effective, there is also another possible explanation that all behavioral studies in this review started the measurement after 30–50 min. Even though CSF levels of oxytocin are already elevated 35 min post-administration, behavioral effects of oxytocin on social behavior in rhesus macaques have been observed after 2 h post-administration (74). Therefore, a lack of positive results might not only mean that oxytocin does not affect behavior, but also that the effects possibly could not yet be measured. However, most of the studies here did not report the task/experiment duration, making it impossible to assess this line of thought. On the *neural level*, only one out of four fMRI studies found an effect of IN-OXY during the anticipatory phase of social reward processing, reporting an increase in VTA activity (63), a reward-related brain area mainly regulated by dopamine (3). Nonetheless, all these fMRI studies used a similar task of low ecological validity, providing a social reward in the form of a stranger’s happy face image. Still, modulation of social reward anticipation by IN-OXY administration is expected, especially in light of the known co-expression of oxytocin receptor gene (OXTR) with various dopaminergic gene sets (75). Overall, both behavioral and fMRI studies did not provide convincing evidence for such an effect.

### 4.2. Social reward consumption

During social reward consumption, studies utilized predominantly the fMRI measurement and to some extent the self-reported measurement. In the self-reported measurement domain, only two (42, 73) out of five studies found an effect (see Table 1). However, conscious reward-related feelings might require additional neural mechanisms, and be undetected by the individual, while unconscious hedonic reactions can still be manifested in behavior and neural processing (3).

In contrast to the self-reports, most fMRI assessments found an effect. The results provide information about the regions that are most commonly modulated by IN-OXY during social reward consumption. The prefrontal cortex, insula, precuneus, ACC, amygdala, and striatum were identified as the six key regions modulated by IN-OXY at least in four fMRI studies (see Table 3). The ventromedial PFC including the OFC has been repeatedly associated with hedonic experiences (3, 76–78), the OFC is believed to signal reward value (79–83), especially, its medial parts (84). Kennerley et al. (85) have studied the role of ACC in reward processing and indicated its role in positive valence-specific reward prediction error coding, with ACC signaling an unexpectedly good reward outcome, but not unexpectedly bad outcome. ACC together with the ventrolateral PFC is further involved in emotion regulation (86). Striatum is an important node in the reward system (3), with its subregions to be dissociable in their contributions to the motivational versus the hedonic component of the affective processing of reward (87). The results on striatum are further in line with research examining human mRNA expression in the brain of the oxytocin receptor (OXTR) and the oxytocin secretion gene (CD38). This research found increased expression of OXTR and CD38 specifically in the caudate and putamen, along with the pallidum, thalamus and the olfactory region (75). It is however worth noting that overall the studies included in this review did not show increased activity in the pallidum, the thalamus nor in the olfactory region, in response to IN-OXY during social reward tasks. Next, previous studies indicated a role of the insula in emotional and salience processing (88–90) as well as its anterior aspect linked to processing of rewards (91), and the precuneus is suggested to be a part of the brain’s social and self-referential circuitry (92). These brain areas are hubs of large-scale neural networks including the salience network, comprising the insula, ACC, and amygdala (93); and default mode network, which involves medial PFC and precuneus. Oxytocin therefore appears to increase the salience of consumed social rewards, self-referential processing and the perceived value of received rewards. Moreover, studies using connectivity measures also found increased coupling of the insula with amygdala (39), the amygdala with regions such as the OFC, precuneus, ACC, and MTG (72), ventral striatum with the amygdala and the precuneus (71), and ventral striatum with the middle frontal gyrus (71). These brain regions entail the so-called hedonic hotspots, which are regulated, among others, by opioidergic activity (3). Interestingly, there is evidence showing a co-expression of OXTR mRNA and the opioid receptor mRNA (10), a finding recently limitedly supported by research on oxytocin pathway gene networks in the human brain (75, 94), suggesting an interaction between the oxytocin and the opioid pathways.

### 4.3. Type of stimuli

It is of interest that during social reward anticipation, studies that found an effect of IN-OXY were using an interactive task of higher ecological validity. This is in line with a study by Declerck et al. (30) reporting that effects of IN-OXY on cooperative behavior were not present when the interaction partner was anonymous. These findings together indicate that interactive tasks with real (or believed to be) people might be more effective in oxytocin studies rather than tasks using simple, i.e., non-interactive/unimodal stimuli with faces of strangers. Regarding reward consumption, the majority of studies, on the other hand, utilized simple stimuli instead of interactive tasks. As reward consumption studies used mainly imaging and the reward anticipation studies used mainly behavioral measures, it may be that overall the potential IN-OXY effects of such doses are detected more easily on the (neuro)physiological level. At any rate, it is possible that utilizing interactive tasks could lead to more valid and robust findings across reward phases and measurement types. However, more data are still needed to specifically test the effects of IN-OXY on social reward processing stemming from any kind of social interaction.

### 4.4. Dosage and remarks on the administration route

Comparing the effects of IN-OXY between different dosages was another aim of this review. However, we cannot provide an answer to the question of whether the higher or lower doses are more effective, as the different dosages appear to be similarly effective in modulating social reward processing. Most of the included studies used the common dose of 24 IU, one of them used age-dependent dosing with the highest dose of 24 IU, and one study used a dose of approximately 26 IU. The six remaining studies used a dose of 40 IU. Nonetheless, the results of studies using different dosages are affected by other methodological aspects, which precludes identifying the effectivity of the doses as such.

This review investigated only studies that administered oxytocin intranasally. The decision was due to the lower side effects and higher efficiency in elevating cerebrospinal cerebrospinal fluid concentrations than the intravenous route of administration (15, 43, 44). Here, several studies suggest that intranasally administered oxytocin primarily exerts its central effects *via* the nose-to-brain route, instead of crossing the blood–brain barrier (47, 95, 96). However, it needs to be mentioned that these studies still do not provide direct confirmation of the nose-to-brain oxytocin administration route. Even though some recent animal studies using novel oxytocin receptor radiotracer demonstrated an increased detection of the radiotracer in the olfactory bulbs of rats after intranasal administration (vs. intravenous administration) (97), a direct confirmation on humans is still lacking mainly due to the lack of an identified human oxytocin ligand (98).

### 4.5. Participants’ sex/gender

We also aimed to examine the sex-differences in effects of IN-OXY on social reward processing. Unfortunately, because of the complicated sex-specific brain responses to IN-OXY, oxytocin research tends to focus mostly on men (89). This is also the case for studies in this review. Most of them were conducted solely on male samples, or included only a small proportion of female participants, so the effects could not be compared between the sexes/genders. Only six studies involved a substantial portion of both men and women in their sample. Of these, only two found significant differences between them. Xu et al. (64) found an effect of IN-OXY only for men, and Ma et al. (39) found that in men social reward processing was increased while in women it was decreased after IN-OXY. These findings are consistent with previous studies reporting increases in reward-related neural processing in men and decreases in women (53, 73, 99, 100). These studies support the sex-dependent inverted U-shaped dose–response hypothesis of oxytocin, first proposed by Rilling et al. (37) and then fully fleshed out by Borland et al. (52), which states that women have higher baseline oxytocin levels in their CSF than men, so exogenous oxytocin administration might overstimulate their oxytocin system, and decrease the reward-related neural activity relative to baseline (37, 52). These findings might suggest that individuals with higher baseline CSF levels of oxytocin, which could also include some patient groups, might need a lower dose of oxytocin to benefit from the treatment. Four out of six studies with samples of mixed male and female participants did not find any difference between the sexes/genders (62, 65, 67, 68) and, additionally, one study conducted solely on a female sample even reported increase in reward-related activity in anticipation (63). This, however, might not stand completely against the hypothesis, because, for example, Nawijn et al. reported that they did not find any baseline (i.e., placebo) differences between the sexes/genders (67), which the studies reporting opposite effects of IN-OXY for women did (39, 53, 100). These findings indicate that there might be other important factors beside the participants’ sex/gender that affect the baseline levels of oxytocin among different individuals.

Three studies investigated the potential changes during the consumption phase in women (33, 70, 72). All of these studies had in common the utilization of the infant stimuli, and two of them assessed also the amygdala reactivity (33, 72). It is of interest that both of these studies, together with the study by Ma et al. report a decreased (and not increased) reactivity of the amygdala in women in response to the IN-OXY treatment (33, 39, 72). This is in contrast to findings in men that systematically showed an upregulation of the amygdala activity (39, 71, 73). These studies together indicate a possibility of a sex-dependent reactivity in response to oxytocin that might be specific to the amygdala. The other explanation may lie in the nature of the stimuli presented. Studies by Bos et al. (33) and Riem et al. (72) both utilized infant stimuli that are generally arousing and induce caretaking motivation tendencies (101). Oxytocin is considered to be the primal neurotransmitter associated with infant caretaking and bond formation (102, 103). At the same time the amygdala generally reacts to salient stimuli (20, 104) and has potentially a high density of oxytocin receptors as shown in other mammals (105). It is thus possible that an already higher concentration of oxytocin in the system due to the IN-OXY administration gives a feedback signal to the amygdala, to no longer signal the salience of the caretaking-relevant stimuli, as the oxytocin implied in caretaking is already upregulated in the system. Nevertheless, more studies are necessary in order to address the relevance of these preliminary contrasting findings in the amygdala.

### 4.6. Onset time

Even though a well calibrated onset (and the subsequent task length) seems to be crucial for maximizing any drug effects, the studies included in this review used various onset times for their experimental task after intranasal oxytocin administration. This was varying from 30 to 50 min and was often without a justification for such a decision. The inconsistent results of the reported studies however do not allow us to draw any conclusions about the efficiency of different utilized onsets. There is no clear consensus about the optimal dose-test latency to allow the greatest concentration of oxytocin in CSF. A study by Spengler et al. (106) used a sample of 116 healthy men and found, that the greatest effectivity of oxytocin was during 45–70 min post-administration. Paloyelis et al. (107) measured cerebral blood flow in 32 healthy men and reported peak response at 39–51 min post-administration. Striepens et al. (43) measured CSF concentrations of oxytocin on a sample of 15 subjects and found that the increase was significant no earlier than after 75 min post-administration. Chang et al. (74) used six rhesus macaques to measure CSF levels of oxytocin and found significant increase after 35 min, however, the prosocial behavioral effects were manifested no earlier than 2 h post-administration. Direct comparison between shorter (e.g., 35 min) and longer (e.g., 2 h) onsets in the IN-OXY effectiveness are, however, still lacking.

### 4.7. Clinical condition

Oxytocin is being studied as a potential treatment for social deficits in various clinical conditions. In the current review we summarized results of seven studies, which attempted to measure the effects of IN-OXY treatment on social reward processing in clinical samples. Overall, four of these studies reported significant effect of oxytocin on social reward processing (38, 40, 67, 71). Some of these studies also included a healthy control group, but most of them did not find a significant difference between the groups, indicating that IN-OXY treatment might be similarly effective for healthy people and patient groups with social deficits. However, the study by Nawijn et al. (67) found that IN-OXY increased social reward processing in PTSD patients and decreased it in healthy controls. Moreover, several of these studies indicate an influence of individual characteristics, for example Wang and Ma (66) report that IN-OXY selectively increased desire to know positive social evaluation (i.e., social reward) in individuals with higher depression scores. Groppe et al. (63) found that behavioral performance on anticipatory social reward was affected by differences in sociability with enhanced performance in individuals scoring low on self-reported measures of agreeableness. Bradley et al. compared the effect of IN-OXY between schizophrenia patients and healthy controls, and even though the difference did not reach statistical significance, the effect was more pronounced in the patient group. Similar findings have been reported previously, indicating that less socially adaptive individuals might benefit the most of oxytocin treatment (38). Bartz et al. suggested that oxytocin can only boost social capacities up to a certain limit beyond which the treatment has not further effect (108). The lack of measurement of individual differences in social proficiency might be one possible explanation for the inconsistent results among previous studies.

### 4.8. Future directions

As the oxytocin studies using simple social stimuli appear to be less effective in modulating social reward processing, future studies should opt for social rewards derived from *interactive* tasks involving real, or believed to be real, interaction partners and social situations that commonly happen in real-world social environments.

Oxytocin research is currently largely investigating male participants while ignoring the necessity of female samples, even though the effects seem to be to a great extent *sex-dependent*. It has been suggested that these sex-differences might be due to differential baseline oxytocin functioning caused by hormonal differences (109, 110); however, as it appears that participants sex/gender might not be the only relevant cause of baseline differences, researchers first need to identify which other individual characteristics might be associated with oxytocin system functioning. One of these might be the *general social abilities* or social adaptivity, which has been reported as a characteristic correlating with the effects of IN-OXY in the previous studies (63, 108). To generate consistent findings, the oxytocin research also needs more *dose–response* studies to identify which doses are the most effective for specific groups of individuals. More information about the most adequate onset necessary for the greatest concentration of oxytocin in CSF is also still lacking. In line with this, it would also be of interest to experimentally explore the appropriate onset-based time window of the possible oxytocin effects to be manifested on the subjective and the behavioral level.

## 5. Limitations

### 5.1. Limitations of studies

Detailed information about limitations for each reported study is provided in the Quality assessment form in the Supplementary material. One limitation which applies especially to the studies using a simple, i.e., unimodal/non-interactive social stimulus is the low external validity, as the design used to elicit social reward was often somewhat artificial and far from real-world social situations.

Further, samples consisted mainly of healthy male participants. It is, however, important to focus on determining whether oxytocin can also improve social reward processing in women in response to various rewards and specific patient groups that display social reward impairments. Additionally, important information such as peak MNI coordinates, exact *p* values, or exact proportion of female participants, were missing in some studies.

Most studies also did not provide enough information about their sampling methods and source population. This could complicate the ability to compare the results of individual studies and it could also indicate a risk of potential bias in results of these studies. Notably, earlier studies with oxytocin might have suffered from being under-powered, and therefore able to detect only a limited range of medium-to-large effect sizes (45). On the other hand, most reported meta-analysis summary effect size estimates are small [i.e., *d* = 0.2 (45)]. Thus, by identifying and utilizing the smallest effect size of interest for sample size estimation, wider range of effect sizes could reliably be detected (98).

### 5.2. Limitations of the review process

Although three major databases and three preprint servers have been used for searches in this review, some relevant papers might have been missed due to not being available in these databases. Next, in contrast to our initial preregistration, we decided to include also studies utilizing infant stimuli, given our re-assessment of their primal relevance as social rewards, and especially in the context of oxytocin manipulation. As the task designs that can measure social reward may be here even more heterogeneous, some relevant studies might have been missed due to lacking keywords we used to create the command lines for database searches. Further studies could have been overlooked due to being written in other languages than English, as only studies written in English have been searched for. Additionally, when studies did not report information about some of the inclusion criteria, we asked the authors for additional information. However, when unreachable by email correspondence, we excluded them from further analysis. Nonetheless, some of these might have, in fact, be eligible but were excluded due to the lack of information. Further, we did not assess publication bias, therefore some studies with insignificant results which were left unpublished may cause additional bias in our results. It is worth noting that out of the original 385 studies first collected and screened only 19 could be included for analysis in this review, suggesting that cumulative evidence is still limited. Lastly, given that the design of this paper was conceptualized as a systematic review and not a meta-analysis, the results obtained from the empirical studies were compared with each other in terms of significance and summarized in a narrative format. We do not provide any quantitative summarization of the data.

## 6. Conclusion

This systematic review summarizes results of randomized double-blind placebo-controlled studies examining the effects of IN-OXY on non-sexual social reward processing in humans, with a special focus on the anticipatory- and consummatory-related reward components. The results suggest that IN-OXY is effective in modulating social reward-related brain activity. Its effects during the consumption of the social reward are exerted likely through modulating activity in brain regions, including the PFC, insula, precuneus, ACC, amygdala, and striatum. In contrast, not much support has been found for the effects of IN-OXY on the anticipatory phase. Future research should focus on female (vs. male) samples to determine the possible sex-differences in the effect of oxytocin, but studies should also measure individual characteristics related to social behavior to address individual differences. Importantly, future research should opt for interactive tasks with real or believed to be real interaction partners, as it appears to be crucial for oxytocin effects.

## Data availability statement

The original contributions presented in the study are included in the article/Supplementary material, further inquiries can be directed to the corresponding authors.

## Author contributions

JK: conceptualization of the study, data obtainment and synthesis, tables and figures preparation, supervision, writing original draft, and review and editing. EV: methodology, data obtainment and synthesis, tables and figures preparation, writing original draft, and review. GS: supervision, writing original draft, and review and editing. All authors contributed to the article and approved the submitted version.

## Footnotes

^1^ https://www.crd.york.ac.uk/prospero/display_record.php?ID=CRD42021278945

^2^Sex/gender are not distinguishable here as we did not assess either or, and thus are used interchangeably.
