# Supplementary material for: The effect of intranasal oxytocin on social reward processing in humans: a systematic review
Source: Front Psychiatry. 2023 Sep 14;14:1244027. doi: 10.3389/fpsyt.2023.1244027 (PMC10536251; doi:10.3389/fpsyt.2023.1244027)
Supplement: Supplementary file 1 [file Data_Sheet_1.docx]

Supplementary Material

# Quality assessment

An adapted version of the Down’s and Black Checklist (58) was used. The checklist included four subscales assessing reporting quality (items 1 to 10), external validity (items 11 to 13), internal validity (statistical and methodological bias; items 14 to 26), and power (item 27). All items were scored as 0 (no, or unable to determine), or 1 (yes), except for item 5, which was scored 0 (no, or unable to determine), 1 (partially), or 2 (yes). Quality rating was ranging therefore between 0 to 28 points. Papers were categorized as having (i) a high (0 to 9 points), average (10 to 19 points), or rather low (20 to 28 points) risk of bias. The quality assessment was performed by one reviewer, and the decision was checked by a second reviewer. Possible discrepancies were resolved through discussion.

The overall risk of bias across all studies was mainly low. Quality assessment ratings ranged from 19 to 24 (M = 21.37) with only one study with an average risk of bias (score=19) and the rest of the studies having a rather low risk (scores >19). Reporting-related items (e.g. clear description of hypotheses and outcomes) were in general appropriately addressed, with eight studies receiving perfect or near-perfect scores. Incomplete reporting mainly included omitting potential adverse events.

Studies in general reported poorly on the external validity (e.g. representativeness of the sample). Internal validity-related items (e.g. use of appropriate statistical tests, accuracy of main outcome measures) were in general well-addressed, with an exception of the item 22 where almost no study scored 1: it was generally not possible to determine whether the participant from the experimental and the control group were recruited over the same time period. In addition, another four studies did not score on item 17. Here it was not possible to determine whether the time period between the drug manipulation and the outcome was the same for the experimental and the control group.

Lastly almost no study adequately reported on their power and only two studies mentioned that they performed an adequate apriori power analysis before collecting the data.

**Supplementary Table 1: Item by Item Scores for Each Individual Study**

|  | Alvares et al., 2010 | Groppe et al., 2013 | Ellingsen et al., 2014 | Scheele et al., 2014 | Hu et al., 2015 | Andari et al., 2016 | Gordon et al., 2016 | Nawijn et al., 2017 | Xu et al., 2017 | Fulford et al., 2018 | Greene et al., 2018 | Ma et al., 2018 | Bradley et al., 2020 | Chen, Li, et al., 2020 | Wang & Ma, 2020 | Mayer et al., 2021 | Riem et al., 2012 | Bos et al., 2018 | Gregory et al., 2015 |
| --- | --- | --- | --- | --- | --- | --- | --- | --- | --- | --- | --- | --- | --- | --- | --- | --- | --- | --- | --- |
| 1 | **1** | **1** | **1** | **1** | **1** | **1** | **1** | **1** | **1** | **1** | **1** | **1** | **1** | **1** | **1** | **1** | **1** | **1** | **1** |
| 2 | **1** | **1** | **1** | **1** | **1** | **1** | **1** | **1** | **1** | **1** | **1** | **1** | **1** | **1** | **1** | **1** | **1** | **1** | **1** |
| 3 | **0** | **1** | **1** | **1** | **1** | **1** | **1** | **1** | **1** | **1** | **1** | **1** | **1** | **1** | **1** | **1** | **1** | **1** | **1** |
| 4 | **1** | **0** | **1** | **1** | **1** | **1** | **1** | **1** | **1** | **1** | **1** | **1** | **1** | **1** | **1** | **1** | **1** | **1** | **1** |
| 5 | **1** | **2** | **2** | **2** | **2** | **2** | **1** | **2** | **2** | **1** | **2** | **2** | **2** | **2** | **2** | **2** | **2** | **2** | **2** |
| 6 | **1** | **1** | **1** | **1** | **1** | **1** | **1** | **1** | **1** | **1** | **1** | **1** | **1** | **1** | **1** | **1** | **1** | **1** | **1** |
| 7 | **1** | **1** | **1** | **1** | **1** | **0** | **1** | **1** | **1** | **1** | **1** | **1** | **1** | **0** | **1** | **1** | **1** | **1** | **1** |
| 8 | **1** | **0** | **0** | **1** | **0** | **0** | **0** | **1** | **0** | **0** | **0** | **0** | **0** | **0** | **0** | **1** | **0** | **0** | **0** |
| 9 | **1** | **1** | **1** | **0** | **1** | **1** | **1** | **1** | **1** | **0** | **1** | **1** | **1** | **1** | **0** | **1** | **1** | **1** | **1** |
| 10 | **1** | **1** | **1** | **1** | **1** | **1** | **0** | **1** | **1** | **1** | **0** | **1** | **1** | **0** | **1** | **1** | **0** | **1** | **1** |
| 11 | **0** | **0** | **0** | **0** | **0** | **0** | **0** | **0** | **0** | **0** | **0** | **0** | **0** | **0** | **0** | **0** | **0** | **0** | **0** |
| 12 | **0** | **0** | **0** | **0** | **0** | **0** | **0** | **0** | **0** | **0** | **0** | **0** | **0** | **0** | **0** | **0** | **0** | **0** | **0** |
| 13 | **1** | **0** | **0** | **0** | **0** | **1** | **0** | **0** | **1** | **1** | **0** | **1** | **1** | **0** | **1** | **0** | **1** | **1** | **0** |
| 14 | **1** | **1** | **1** | **1** | **1** | **1** | **1** | **1** | **1** | **1** | **1** | **1** | **1** | **1** | **1** | **1** | **1** | **1** | **1** |
| 15 | **1** | **1** | **1** | **1** | **1** | **1** | **1** | **1** | **1** | **1** | **1** | **1** | **1** | **1** | **1** | **1** | **1** | **1** | **1** |
| 16 | **1** | **1** | **1** | **1** | **1** | **1** | **1** | **1** | **1** | **1** | **1** | **1** | **1** | **1** | **1** | **1** | **1** | **1** | **1** |
| 17 | **1** | **1** | **0** | **1** | **1** | **1** | **0** | **0** | **1** | **1** | **0** | **1** | **1** | **1** | **1** | **1** | **1** | **1** | **1** |
| 18 | **1** | **1** | **1** | **1** | **1** | **1** | **1** | **1** | **1** | **1** | **1** | **1** | **1** | **1** | **1** | **1** | **1** | **1** | **1** |
| 19 | **1** | **1** | **1** | **1** | **1** | **1** | **1** | **1** | **1** | **1** | **1** | **1** | **1** | **1** | **1** | **1** | **1** | **1** | **1** |
| 20 | **1** | **1** | **1** | **1** | **1** | **1** | **1** | **1** | **1** | **1** | **1** | **1** | **1** | **1** | **1** | **1** | **1** | **1** | **1** |
| 21 | **1** | **0** | **1** | **1** | **0** | **1** | **1** | **1** | **1** | **1** | **1** | **0** | **0** | **1** | **1** | **1** | **1** | **1** | **0** |
| 22 | **0** | **0** | **0** | **0** | **0** | **0** | **0** | **0** | **0** | **0** | **0** | **0** | **0** | **0** | **0** | **0** | **1** | **0** | **0** |
| 23 | **1** | **1** | **1** | **1** | **1** | **1** | **1** | **1** | **1** | **1** | **1** | **1** | **1** | **1** | **1** | **1** | **1** | **1** | **1** |
| 24 | **1** | **1** | **1** | **1** | **1** | **1** | **1** | **1** | **1** | **1** | **1** | **1** | **1** | **1** | **1** | **1** | **1** | **0** | **1** |
| 25 | **1** | **1** | **1** | **1** | **1** | **0** | **1** | **1** | **1** | **1** | **1** | **1** | **1** | **1** | **1** | **1** | **1** | **1** | **1** |
| 26 | **1** | **1** | **1** | **0** | **1** | **1** | **1** | **1** | **1** | **0** | **1** | **1** | **1** | **1** | **0** | **1** | **1** | **1** | **1** |
| 27 | **0** | **0** | **0** | **0** | **0** | **0** | **0** | **0** | **0** | **0** | **0** | **0** | **0** | **0** | **1** | **1** | **0** | **0** | **0** |

**Supplementary Table 2: Overall Score for Each Individual Study**

|  | Alvares et al., 2010 | Groppe et al., 2013 | Ellingsen et al., 2014 | Scheele et al., 2014 | Hu et al., 2015 | Andari et al., 2016 | Gordon et al., 2016 | Nawijn et al., 2017 | Xu et al., 2017 | Fulford et al., 2018 | Greene et al., 2018 | Ma et al., 2018 | Bradley et al., 2020 | Chen, Li, et al., 2020 | Wang & Ma, 2020 | Mayer et al., 2021 | Riem et al., 2012 | Bos et al., 2018 | Gregory et al., 2015 |
| --- | --- | --- | --- | --- | --- | --- | --- | --- | --- | --- | --- | --- | --- | --- | --- | --- | --- | --- | --- |
| Reporting | 9 | 9 | 10 | 10 | 10 | 9 | 8 | 11 | 10 | 8 | 9 | 10 | 10 | 8 | 9 | 11 | 9 | 10 | 10 |
| External validity | 1 | 0 | 0 | 0 | 0 | 1 | 0 | 0 | 1 | 1 | 0 | 1 | 1 | 0 | 1 | 0 | 1 | 1 | 0 |
| Internal validity | 12 | 11 | 11 | 11 | 11 | 11 | 11 | 11 | 12 | 11 | 11 | 11 | 11 | 12 | 11 | 12 | 13 | 11 | 11 |
| Power | 0 | 0 | 0 | 0 | 0 | 0 | 0 | 0 | 0 | 0 | 0 | 0 | 0 | 0 | 1 | 1 | 0 | 0 | 0 |
| Overall | 22 | 20 | 21 | 21 | 21 | 21 | 19 | 22 | 23 | 20 | 20 | 22 | 22 | 20 | 22 | 24 | 23 | 22 | 21 |

# Search Strategy

**Web of Science**

TS=(oxytocin*) AND TS=(intranasal*) AND TS=(*social* OR affilia*) AND TS=(reward* OR wanting OR incentiv* OR (pursuit AND goal*) OR (motivat* AND salien*) OR desir* OR liking OR (hedonic AND impact) OR (hedonic AND value) OR (hedonic AND react*) OR pleasur* OR (approach* AND motivat*))

**PubMed**

((oxytocin*[Title/Abstract]) AND (intranasal*[Title/Abstract]) AND ((*social*[Title/Abstract]) OR (affilia*[Title/Abstract])) AND ((reward*[Title/Abstract]) OR (wanting[Title/Abstract]) OR (incentiv*[Title/Abstract]) OR (pursuit AND goal*[Title/Abstract]) OR (motivat* AND salien*[Title/Abstract]) OR (desir*[Title/Abstract]) OR (liking[Title/Abstract]) OR (hedonic AND impact[Title/Abstract]) OR (hedonic AND value[Title/Abstract]) OR (hedonic AND react*[Title/Abstract]) OR (pleasur*[Title/Abstract]) OR (approach* AND motivat*[Title/Abstract])))

**Scopus**

TITLE-ABS-KEY ( oxytocin* )  AND  TITLE-ABS-KEY ( intranasal* )  AND  TITLE-ABS-KEY ( *social*  OR  affilia*)  AND  TITLE-ABS-KEY ( reward*  OR  wanting  OR  incentiv*  OR  ( pursuit  AND  goal* )  OR  ( motivat*  AND  salien* )  OR  desir*  OR  liking  OR  ( hedonic  AND  impact )  OR  ( hedonic  AND  value)  OR  ( hedonic  AND  react* )  OR  pleasur*  OR  ( approach*  AND  motivat* ))

**BioRxiv, MedRxiv, PsyArXiv**

intranasal AND oxytocin AND social AND reward

**3 Supplementary Table: PRISMA 2020 Checklist**

| **Section and Topic** | **Item #** | **Checklist item** | **Item reported** |
| --- | --- | --- | --- |
| **TITLE** | | |  |
| Title | 1 | Identify the report as a systematic review. | Yes |
| **ABSTRACT** | | |  |
| Abstract | 2 | See the PRISMA 2020 for Abstracts checklist. |  |
| **INTRODUCTION** | | |  |
| Rationale | 3 | Describe the rationale for the review in the context of existing knowledge. | Yes |
| Objectives | 4 | Provide an explicit statement of the objective(s) or question(s) the review addresses. | Yes |
| **METHODS** | | |  |
| Eligibility criteria | 5 | Specify the inclusion and exclusion criteria for the review and how studies were grouped for the syntheses. | Yes |
| Information sources | 6 | Specify all databases, registers, websites, organisations, reference lists and other sources searched or consulted to identify studies. Specify the date when each source was last searched or consulted. | Yes |
| Search strategy | 7 | Present the full search strategies for all databases, registers and websites, including any filters and limits used. | Yes |
| Selection process | 8 | Specify the methods used to decide whether a study met the inclusion criteria of the review, including how many reviewers screened each record and each report retrieved, whether they worked independently, and if applicable, details of automation tools used in the process. | Yes |
| Data collection process | 9 | Specify the methods used to collect data from reports, including how many reviewers collected data from each report, whether they worked independently, any processes for obtaining or confirming data from study investigators, and if applicable, details of automation tools used in the process. | Yes |
| Data items | 10a | List and define all outcomes for which data were sought. Specify whether all results that were compatible with each outcome domain in each study were sought (e.g. for all measures, time points, analyses), and if not, the methods used to decide which results to collect. | Yes |
|  | 10b | List and define all other variables for which data were sought (e.g. participant and intervention characteristics, funding sources). Describe any assumptions made about any missing or unclear information. | N/A |
| Study risk of bias assessment | 11 | Specify the methods used to assess risk of bias in the included studies, including details of the tool(s) used, how many reviewers assessed each study and whether they worked independently, and if applicable, details of automation tools used in the process. | Yes |
| Effect measures | 12 | Specify for each outcome the effect measure(s) (e.g. risk ratio, mean difference) used in the synthesis or presentation of results. | Yes |
| Synthesis methods | 13a | Describe the processes used to decide which studies were eligible for each synthesis (e.g. tabulating the study intervention characteristics and comparing against the planned groups for each synthesis (item #5)). | Yes |
|  | 13b | Describe any methods required to prepare the data for presentation or synthesis, such as handling of missing summary statistics, or data conversions. | Yes |
|  | 13c | Describe any methods used to tabulate or visually display results of individual studies and syntheses. | N/A |
|  | 13d | Describe any methods used to synthesize results and provide a rationale for the choice(s). If meta-analysis was performed, describe the model(s), method(s) to identify the presence and extent of statistical heterogeneity, and software package(s) used. | Yes |
|  | 13e | Describe any methods used to explore possible causes of heterogeneity among study results (e.g. subgroup analysis, meta-regression). | N/A |
|  | 13f | Describe any sensitivity analyses conducted to assess robustness of the synthesized results. | N/A |
| Reporting bias assessment | 14 | Describe any methods used to assess risk of bias due to missing results in a synthesis (arising from reporting biases). | Yes |
| Certainty assessment | 15 | Describe any methods used to assess certainty (or confidence) in the body of evidence for an outcome. | N/A |
| **RESULTS** | | |  |
| Study selection | 16a | Describe the results of the search and selection process, from the number of records identified in the search to the number of studies included in the review, ideally using a flow diagram. | Yes |
|  | 16b | Cite studies that might appear to meet the inclusion criteria, but which were excluded, and explain why they were excluded. | N/A |
| Study characteristics | 17 | Cite each included study and present its characteristics. | Yes |
| Risk of bias in studies | 18 | Present assessments of risk of bias for each included study. | Yes |
| Results of individual studies | 19 | For all outcomes, present, for each study: (a) summary statistics for each group (where appropriate) and (b) an effect estimate and its precision (e.g. confidence/credible interval), ideally using structured tables or plots. | Yes |
| Results of syntheses | 20a | For each synthesis, briefly summarise the characteristics and risk of bias among contributing studies. | Yes |
|  | 20b | Present results of all statistical syntheses conducted. If meta-analysis was done, present for each the summary estimate and its precision (e.g. confidence/credible interval) and measures of statistical heterogeneity. If comparing groups, describe the direction of the effect. | N/A |
|  | 20c | Present results of all investigations of possible causes of heterogeneity among study results. | N/A |
|  | 20d | Present results of all sensitivity analyses conducted to assess the robustness of the synthesized results. | N/A |
| Reporting biases | 21 | Present assessments of risk of bias due to missing results (arising from reporting biases) for each synthesis assessed. | Yes |
| Certainty of evidence | 22 | Present assessments of certainty (or confidence) in the body of evidence for each outcome assessed. | N/A |
| **DISCUSSION** | | |  |
| Discussion | 23a | Provide a general interpretation of the results in the context of other evidence. | Yes |
|  | 23b | Discuss any limitations of the evidence included in the review. | Yes |
|  | 23c | Discuss any limitations of the review processes used. | Yes |
|  | 23d | Discuss implications of the results for practice, policy, and future research. | Yes |
| **OTHER INFORMATION** | | |  |
| Registration and protocol | 24a | Provide registration information for the review, including register name and registration number, or state that the review was not registered. | Yes |
|  | 24b | Indicate where the review protocol can be accessed, or state that a protocol was not prepared. | Yes |
|  | 24c | Describe and explain any amendments to information provided at registration or in the protocol. | Yes |
| Support | 25 | Describe sources of financial or non-financial support for the review, and the role of the funders or sponsors in the review. | N/A |
| Competing interests | 26 | Declare any competing interests of review authors. | Yes |
| Availability of data, code and other materials | 27 | Report which of the following are publicly available and where they can be found: template data collection forms; data extracted from included studies; data used for all analyses; analytic code; any other materials used in the review. | N/A |

*From:*  Page MJ, McKenzie JE, Bossuyt PM, Boutron I, Hoffmann TC, Mulrow CD, et al. The PRISMA 2020 statement: an updated guideline for reporting systematic reviews. BMJ 2021;372:n71. doi: 10.1136/bmj.n71 For more information, visit: <http://www.prisma-statement.org/>
